# Semantic, syntactic, and phonological processing of written words in adult developmental dyslexic readers: an event-related brain potential study

**DOI:** 10.1186/1471-2202-8-52

**Published:** 2007-07-17

**Authors:** Jascha Rüsseler, Petra Becker, Sönke Johannes, Thomas F Münte

**Affiliations:** 1 Department of Psychology II, Neuropsychology Unit, Otto-von-Guericke University Magdeburg, Germany; 2 Reha-Klinik Bellikon, Switzerland

## Abstract

**Background:**

The present study used event-related brain potentials to investigate semantic, phonological and syntactic processes in adult German dyslexic and normal readers in a word reading task. Pairs of German words were presented one word at a time. Subjects had to perform a semantic judgment task (house – window; are they semantically related?), a rhyme judgment task (house – mouse; do they rhyme?) and a gender judgment task (das – Haus [the – house]; is the gender correct? [in German, house has a neutral gender: das Haus]).

**Results:**

Normal readers responded faster compared to dyslexic readers in all three tasks. Onset latencies of the N400 component were delayed in dyslexic readers in the rhyme judgment and in the gender judgment task, but not in the semantic judgment task. N400 and the anterior negativity peak amplitudes did not differ between the two groups. However, the N400 persisted longer in the dyslexic group in the rhyme judgment and in the semantic judgment tasks.

**Conclusion:**

These findings indicate that dyslexics are phonologically impaired (delayed N400 in the rhyme judgment task) but that they also have difficulties in other, non-phonological aspects of reading (longer response times, longer persistence of the N400). Specifically, semantic and syntactic integration seem to require more effort for dyslexic readers and take longer irrespective of the reading task that has to be performed.

## Background

Developmental dyslexia is characterized by a difficulty in written language processing in persons possessing the intelligence and motivation considered necessary for accurate and fluent reading. More formally, it has been defined as a specific developmental impairment in the ability to read and spell despite adequate educational resources, average non-verbal intelligence, no obvious sensory deficits and appropriate socio-cultural opportunities [1,2]. Dyslexia occurs in all languages and is perhaps the most common developmental learning disorder affecting children with prevalence rates ranging from 5% to 17.5% [3-6]. Both, prospective and retrospective longitudinal studies indicate that dyslexia is a chronic, persistent condition [7,8] and, thus, does not represent a developmental lag [9]. Over time, poor and good readers tend to maintain their relative positions along the spectrum of reading ability. However, adult dyslexic readers have problems that differ from those of dyslexic children. Their main problems are poor spelling, slow reading, decoding, and nonword-reading [10-12].

Most researchers agree that developmental dyslexia is a disorder of neurobiological origin. Family history is one important risk factor: 23 to 65 percent of children with a dyslexic parent are also dyslexic [13]. Independent research groups identified gene-loci on chromosomes 2, 6, and 15 that appear to be linked to dyslexia [for reviews, see [14-17]], whereas loci on chromosomes 1, 3, and 18 have been reported by single research groups only [18]. The exact relationship between biological and cognitive factors causing the disorder is still under debate, however [for one recent proposal, see [19]]. Theories proposed to explain developmental dyslexia include the visual theory [20], the rapid auditory processing theory [21-23], the cerebellar theory [24], the magnocellular theory [25] and the phonological theory [2,26-29].

A great number of behavioral and event-related brain potential (ERP) studies have been aimed at seeking evidence for these theories and thus have used rather simple stimuli [e.g. [30-40]]. The question to what extent different aspects of language processing are impaired has been addressed to a much lesser extent. Therefore, the present study investigates the processing of visually presented pairs of words by means of ERPs in three different conditions: a phonological or rhyme judgment task (RJT), a semantic judgment task (SJT), and a syntactic judgment task (GJT; gender judgment task).

ERPs can be used to study language processing [for reviews, see [41,42]] because different components have been found to be sensitive to semantic and syntactic aspects of language processing. For example, the N400 component of the ERP is a negative peak usually occurring approximately 400 ms post-stimulus. It is generally reduced for visually or auditorily presented words that are semantically primed, i.e. for target words that are preceded by a related word, or that are part of a semantically congruous sentence ([43]; for a review, see [42]). The prevailing interpretation of the N400 is that its amplitude varies as a function of the ease with which a word can be integrated into the overall meaning representation that is built up on the basis of the preceding language input, be it a sentence or a single word [44-48]. In line with this interpretation, N400-amplitude has been found to be influenced by phonological information as well [49-51]. As an example, Rugg [52,53] observed that when the second of two visually presented words rhymed with the first, the N400 for the second word was reduced compared to a non-rhyming word. The scalp distribution of this N400-effect for rhyming words is similar to that of the semantic priming effect. Typically, the N400 has a broad scalp distribution with a slight lateralization to the right and a maximum at centro-parietal locations [54,55].

Syntactic violations have been reported to elicit negativities that differ in timing and scalp distribution from those observed for semantic incongruencies (55, 56]. These negativities usually show a more frontal maximum compared to the N400 and are sometimes larger over the left hemisphere, although in many studies bilateral distributions have been observed [e.g. [45]]. They occur within the same time-range as the N400 (i.e. 300 to 500 ms after stimulus presentation) [57].

ERP-studies of semantic and phonological processing in dyslexic readers have yielded mixed results. Some authors reported delayed, reduced or absent N400 word priming effects for adult [58-60] and adolescent dyslexics [61,62]. These findings were interpreted as reflecting problems in the engagement of long-term semantic memory. These deficits have been found to vary as a function of presentation rate (pronounced N400 differences only at slow presentation rates in a word-by-word sentence reading task) [63], word frequency (more pronounced N400 amplitude reduction for low frequency words) [58], mode of stimulus presentation (visual or auditory), and subtype of reading disability (phonetic dyslexics do not show a normal N400 priming effect for auditorily presented words, but dysphonetic dyslexics do) [61]. In contrast, Bonte and Blomert (2004), Silva-Pereyra et al. (2003), and Rüsseler et al. (2003) [64-66] observed normal N400 priming effects in dyslexic and poor reading children and adults, respectively.

Syntactic processing in dyslexia has not often been the subject of investigation with ERPs, and all of the studies to date have employed sentence reading tasks. Leikin [67] found higher amplitudes and longer latencies for the P200, P300 and P600 in dyslexic readers that were interpreted as suggesting the existence of a syntactic processing weakness in dyslexic readers. Rispens [68] obtained no differences between normal and dyslexic readers in the ELAN, an early, left frontal negativity reflecting a highly automatised processing phase of syntactic parsing [69]. As in the aforementioned studies of Leikin and coworkers, the P600 was changed in dyslexics, but only for some forms of grammatical violations. A recent study by Sabisch and co-workers [70] presented phrase structure violations in passive sentences to dyslexic children and controls. While control children showed an early starting bilaterally distributed anterior negativity and a late centro-parietal positivity (P600), dyslexics ERPs were characterized by a delayed left lateralized anterior negativity, followed by a P600. The authors concluded that early automatic phrase structure building processes, which are reflected in the anterior negativity, are delayed in dyslexic children. Furthermore, the bilateral distribution of the early effect in controls was taken to suggest an involvement of prosodic processes localized in the right hemisphere in addition to the left hemispheric syntactic processes. The left-lateralized negativity in dyslexic on the other hand was interpreted in the sense of phonological impairment in dyslexic children (i.e. no right hemisphere contribution) which might lead to an impariment of syntactic processes. Thus, there is evidence for syntactic processing difficulties in dyslexic readers, but the exact nature of the problems is still a matter of debate.

The heterogenity of the findings in ERP-research on semantic, phonological, and syntactic processing in dyslexia can be attributed to several factors. First, different tasks have been used (word recognition; picture-word-priming; sentence reading; visual or auditory stimulus presentation). Second, dyslexics taking part in the above mentioned studies differed with respect to age and level of reading impairment. Finally, semantic, phonological and syntactic processing have not been studied in the same sample of dyslexics outside of the sentence context. Thus, in the present study, semantic, phonological and syntactic processing was investigated in a same sample of adult normal and dyslexic readers. To this purpose, we used the presentation of word pairs rather than sentences.

As stated earlier, three word reading tasks were employed. In the RJT, subjects had to decide whether the second of two consecutively presented words rhymed with the first. In the SJT, participants had to judge whether the second word was semantically related to the first whereas in the GJT, it had to be decided whether the gender of the critical word matched that indicated by the first word. ERPs were computed relative to the presentation of the second word of the consecutively presented word pairs.

We decided to include only high-achieving dyslexic readers (university students) in our sample to minimize the chances of studying individuals with another comorbid developmental disorder, such as specific language impairment (SLI), ADHD or developmental coordination disorder [for a similar approach, see [71]]. Thus, any deficit in language processing found in the present study can be viewed as a "core deficit" of developmental dyslexia.

Because of the well-known difficulties in phonological processing in dyslexia [2,26-29], we expected a delay of ERP effects in the RJT. To the extent that these difficulties might propagate to later processing stages (see, for example, [70]), a delay in the ERP effects to gender and semantic violations was also expected.

## Results

### Behavioral data

The reaction times (RTs) and percentage of correct responses for the three tasks are shown in Table 1. There were main effects of GROUP (F(1,20) = 27.41, p < .00001) and TASK (F(2,40) = 16.31, p < .00001) reflecting the fact that RTs were fastest in the rhyme judgment task and that normal readers responded faster than dyslexics. No significant interaction between factors GROUP and TASK was obtained. Statistical analysis of the accuracy data revealed the same pattern as the RT analysis indicating that no speed-accuracy trade-off was present for one of the groups.

**Table 1:** Behavioral data

	**RT (ms)**			**% correct responses**		
	**RJT**	**SJT**	**GJT**	**RJT**	**SJT**	**GJT**

**Controls**	1103 (41.8)	1273 (37.4)	1229 (34.1)	97.7 (0.55)	96.1 (0.51)	89.1 (2.42)
**Dyslexics**	1327 (64.2)	1568 (62.7)	1591 (60.1)	93.4 (2.01)	90.7 (2.85)	84.6 (3.64)

Reacton times and percentage of correct responses (s.e.m.) for normal and dyslexic readers in the three tasks.

### Event-related brain potentials

#### RJT

**Figure 1:**
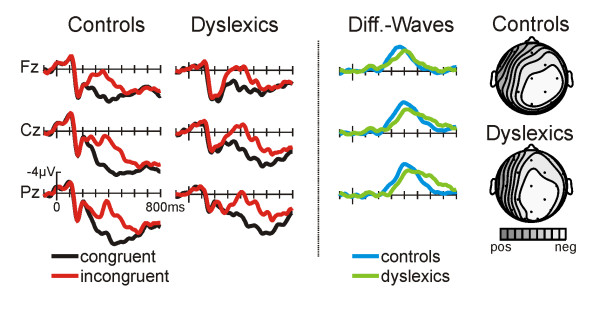
**ERPs in the RJT**. ERPs in the rhyme judgment task for the control groups (left) and for dyslexic readers (middle). The right column depicts the difference waves (incongruent – congruent) for the control group (solid line) and for the dyslexic readers (dotted line). Voltage and time scales of the left column pertain to all columns. Distributions are illustrated for the maximum of the difference waves as spline-interpolated isovoltage maps (relative scaling, controls min/max -5.9/-0.4 μV, dyslexics -4.8/0.2 μV).

For the first 200 ms the ERPs to congruent and incongruent words are almost identical. However, beginning approximately 250 ms after the onset of the word the ERPs to incongruent, non-rhyming words display a more negative-going waveform (see Fig. 1). This effect is most prominent at central and parietal electrode locations and slightly larger for right hemisphere sites (see topographic isovoltage maps derived from the difference waves, figure 1, rightmost column). The incongruency effect is reflected by the difference waves (figure 1, third column) for normal and dyslexic readers. Obviously, the N400 starts and peaks earlier in normal compared to dyslexic readers. By contrast, the N400 persists much longer in dyslexics. Statistically, this is reflected in an earlier N400 onset latency for normal compared to dyslexic readers (one-sided t-test against 0: control 240 ms, dyslexics: 272 ms; measured at electrode C4) and an earlier N400 peak latency (401 ms vs. 493 ms, F(1,20) = 7.14, p = .0146, measured at C4).

Due to the different N400 peak latencies, amplitude differences between normal and dyslexic readers were analyzed using the N400 peak amplitude values. The negative maximum for each subject was determined in a time-window ranging from 200 – 700 ms. In contrast to the visual impression (Fig. 1, third columns), no significant N400 amplitude difference between the groups emerged at any electrode. The analysis of the differences waves yielded the same pattern of results.

The incongruency effect (larger negativity for non-rhymes compared to rhymes) turned out to be highly significant for both, dyslexic and normal readers (see Table 2).

**Table 2:** Statistical analysis of the N400 effect

	**RJT**		**SJT**		**GJT**	
**Electrode(s)**	**controls**	**dyslexics**	**controls**	**dyslexics**	**controls**	**Dyslexics**

**Midline**	50.01	44.03	26.21	11.74	18.15	7.59
	p < .00001	p = .0001	p = .0005	P = .0065	p = .0017	p = .0203
**parasagittal**	32.54	27.39	20.93	9.02	29.79	10.2
	p = .0002	p = .0004	p = .001	P = .0133	p = .0003	p = .0096
**temporal**	13.76	5.04	7.69	5.02	21.88	11.86
	p = .0040	p = .0487	p = .0197	P = .049	p = .0009	p = .0063
**C4**	55.04	48.99	29.02	8.91	21.34	14.06
	p < .00001	p = .0002	p = .0003	p = .0137	p = .001	p = .0038
	6.16 μV vs. 2.25 μV	3.12 μV vs. 0.14 μV	3.32 μV vs. -1.25 μV	2.86 μV vs. -2.22 μV	5.64 μV vs. 2.99 μV	3.29 μV vs. 1.46 μV

Statistical evaluation of the N400 (RJT, SJT) and the anterior negativity (GJT). The F(1,10)- and p-values for the main effect of WORD TYPE (congruent vs. incongruent) are shown for the different electrode clusters used (see text). As a further illustration, the results for the single electrode C4 are also shown. Note that in all analyses except for the midline electrodes, the WORD TYPE by ELECTRODE interactions were significant (all p < .05). Mean amplitudes in the following time-windows were used: RJT: 250 – 600 ms; SJT: 250 – 600 ms; GJT: 450 – 700 ms.

#### SJT

**Figure 2:**
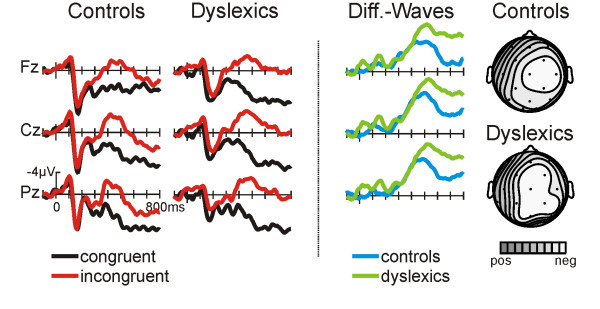
**ERPs in the SJT**. ERPs in the semantic judgment task for the control groups (left) and for dyslexic readers (middle). The right column depicts the difference waves (incongruent – congruent) for the control group (solid line) and for the dyslexic readers (dotted line). Voltage and time scales of the left column pertain to all columns. Distributions are illustrated for the maximum of the difference waves as spline-interpolated isovoltage maps (relative scaling, controls min/max -6.3/-0.3 μV, dyslexics -8.2/2.2 μV).

For the first 200 ms the ERPs to words following semantically congruent primes and to words following incongruent primes are virtually identical. However, beginning approximately 250 ms after the onset of the word, the ERPs to incongruent words display a more negative-going waveform (see Fig. 2, columns 1 and 2). This effect is most prominent at central and parietal electrode locations and again is slightly larger for right hemisphere sites (see topographic isovoltage maps in figure 2). The incongruent – congruent difference waves (figure 2, third column) suggest that the N400 has the same onset latency for normal and dyslexic readers but that it is more extended in time in the dyslexic group. Indeed, N400-onset-latency does not differ between dyslexic and normal readers (one-sided t-test against 0: control 272 ms, dyslexics: 272 ms; measured at electrode C4). However, while the N400 amplitude seems to be larger in the dyslexic group no significant group differences were obtained for the mean amplitude in the 500 to 650 ms and 650 to 800 ms time windows (all p < .20). In addition, no GROUP by ANTERIORITY or GROUP by LATERALITY interactions were seen. Again, the incongruency effect (larger N400 amplitude for incongruent words) turned out to be highly significant for both groups (see Table 2).

An analysis of the mean amplitude values of the difference waves at electrode P4 revealed a strong tendency for the N400 to persist longer in dyslexics compared to the controls (main effect GROUP, 500 – 800 ms, F(1,20) = 3.55, p < .074).

#### GJT

**Figure 3:**
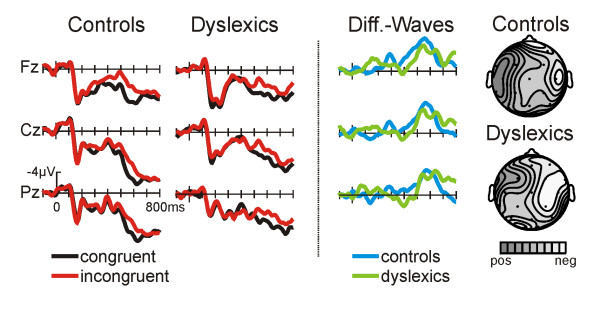
**ERPs in the GJT**. ERPs in the syntactic judgment task for the control groups (left) and for dyslexic readers (middle). The right column depicts the difference waves (incongruent – congruent) for the control group (solid line) and for the dyslexic readers (dotted line). Voltage and time scales of the left column pertain to all columns. Distributions are illustrated for the maximum of the difference waves as spline-interpolated isovoltage maps (relative scaling, controls min/max -3.9/-1.0 μV, dyslexics -3.2/0.3 μV).

For the first 400 ms the ERPs to syntactically correct words and to syntactically incorrect words show a very similar waveform. However, beginning at approximately 400 ms the ERPs to grammatically incorrect words (with respect to the preceding article) display a more negative going waveform (see Fig. 3). This effect is most prominent at central and right temporal electrode locations as is evident in the topographic isovoltage maps (figure 3, rightmost column).

The difference waves (incorrect – correct, figure 3, third column) show an anterior negativity which starts earlier in normal compared to dyslexic readers. This is reflected by onset latency differences between the two groups (one-sided t-test against 0: control 440 ms, dyslexics: 504 ms; measured at electrode C4). Due to the differences in onset latency of the anterior negativity, peak amplitude for each subject was determined in a time-window ranging from 200 to 700 ms to evaluate amplitude differences between the groups. Despite the impression of a slightly larger peak amplitude for the control group, the ANOVAs did not show any significant main effect of GROUP or a significant interaction between the GROUP and the electrodes factors. The incongruency effect (larger anterior negativity amplitude for syntactically incorrect words compared to syntactically correct words) turned out to be highly significant for both groups (see Table 2).

#### Comparison between the three tasks

**Figure 4:**
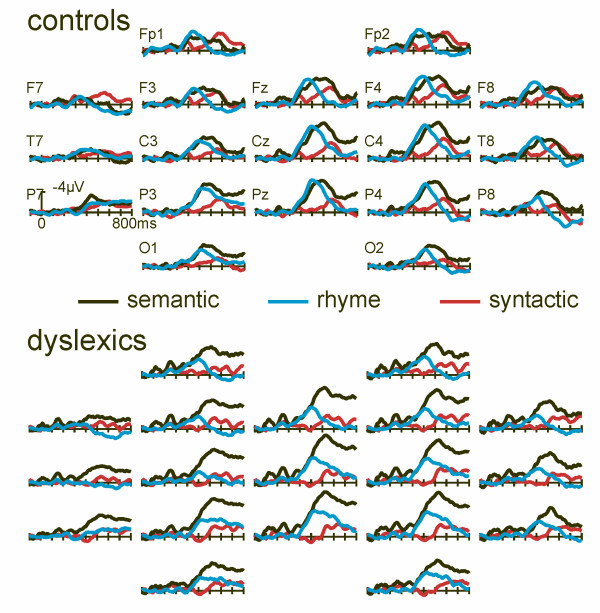
**Difference ERPs for the three tasks**. Difference potentials (incongruent – congruent) for all three tasks for the control group (top) and dyslexic readers (bottom). Note the different onset latencies in the N400 and the anterior negativity both within and across groups.

Figure 4 shows the difference waves of the three tasks for the control group and dyslexic readers. It can be seen that the onset of the difference waves in normal readers does not differ for the semantic and rhyme judgment tasks whereas the anterior negativity for the syntactic judgment task has a later onset latency (controls: RJT: 240 ms; SJT: 272 ms; GJT: 440 ms as determined by serial t-tests at electrode C4). For dyslexic readers, the same pattern emerged, but the incongruency effect for the RJT begins later than for the control group (dyslexics: SJT: 272 ms; RJT: 272 ms; GJT: 504 ms).

The topographies of the incongruency effects can be appreciated from the maps presented in figures 1 to 3. The topographies for rhyme and semantic tasks are very similar. In contrast, the anterior negativity in the GJT shows a more frontal distribution for both groups. Distributions are very similar for both groups. Statistically, this is reflected in non-significant GROUP (dyslexics, control) by ELECTRODE (29) interactions in ANOVAs of the standardized mean amplitudes in the N400/anterior negativity time-windows (RJT: 380–440 ms; SJT: 480–540 ms; GJT: peak GJT: peak amplitude; all F's < 1). Furthermore, in ANOVAs of the standardized data comprising the repeated measures factors TASK (RJT, SJT, GJT) and ELECTRODE (29), significant interactions emerged for TASK and ELECTRODE (controls: F(2,56) = 15.61, p < .0001; dyslexics: F(2,56) = 7.74, p < .002). Post-hoc comparisons revealed that the GJT-anterior negativity topography differs from both, the SJT and the RJT topography of the N400 in dyslexics as well as controls. No differences were seen between SJT and RJT.

## Discussion

The present study employed phonological, semantic, and syntactic judgment tasks to investigate the processing of visually presented words in adult dyslexic and normal readers with event-related brain potentials. Normal readers responded faster compared to dyslexics in all three tasks. Furthermore, both, dyslexic as well as normal readers were faster in their responses in the rhyme judgment compared to the other two tasks. In all three tasks the incongruent words were characterized by more negative going ERPs of different distributions. The onset latencies of these negative effects were delayed in dyslexic readers compared to controls in the phonological judgment and in the gender judgment task, but not in the semantic judgment task. Peak amplitudes did not differ between the two groups. In the phonological judgment and the semantic judgment tasks the incongruency effects persisted longer in the dyslexic groups. While some authors [72,73] have pointed out that the phonological and semantic effects have different distributions, we will use the label N400 for both, the semantic and phonological incongruency effects, while the syntactic effect will be termed anterior negativity.

The phonological N400 displayed a delayed onset and persisted longer in our sample of adult dyslexic readers. This indicates that adult dyslexics have difficulties with phonological processing of written words, a finding that is compatible with the notion of a phonological core deficit in developmental dyslexia [6,74,75]. In line with previous research [64-66], we observed a normal N400 semantic priming effect in adult dyslexic readers: N400 onset latency was not delayed for dyslexics in the semantic judgment task, and N400 peak amplitude was comparable in impaired and normal readers. As was the case with the phonological task, the semantic N400 persisted longer in dylexics. Furthermore, they needed longer to perform the semantic judgment task. Thus, it seems that dyslexic readers need more time and effort for semantic integration processes. Similar results were obtained in a sentence reading task using slow presentation rates comparable to the ones used here (600 ms SOA in the present study, 700 ms SOA in [63]).

With regard to syntactic processing, we observed a delayed onset and a longer persistence of the anterior negativity as well as prolonged response times indicating syntactic processing difficulties in dyslexic adults. Interestingly, in an auditory sentence comprehension task in dyslexic children [70] a delayed anterior negativity was observed as well. Moreover, in this study normal readers showed a bilateral distribution of the negativity (suggesting a contribution of prosodic right hemisphere processes), while the strong left lateralization in the dyslexics suggested no contribution of prosodic/phonological processes in the detection of the phrase structure violations. This pattern might be taken to suggest an impairment of phonological processing which results in problems of automatic syntactic processing. Our own data lend themselves to similar interpretation.

To summarize, the present experiment suggest that adult dyslexic readers do not only show phonological difficulties in written word processing but also have problems in syntactic and semantic integration processes. Remediation programs created for dyslexic adult readers should take these processing deficits into account.

## Conclusion

The present study replicates the well-known finding that dyslexics are phonologically impaired (delayed N400 in the rhyme judgment task). Furthermore, we show that they also have difficulties in other, non-phonological aspects of reading (longer response times, longer persistence of the N400). Specifically, semantic and syntactic integration seem to require more effort for dyslexic readers and take longer irrespective of the reading task that has to be performed. These aspects of reading impairment in dyslexia should be taken into account when designing new interventions for dyslexic readers.

## Methods

### Subjects

Developmental dyslexics were selected from 102 adults with self-reported reading and spelling difficulties who responded to a newspaper advertisement. Out of this collective, all adults above the age of 18 and below 40 with a high-school degree (or attending the final high-school class) were invited for neuropsychological testing, which included full-scale IQ testing by the German version of the WAIS-R (HAWIE-R, [76], a standardized test of spelling skill (R-T; dictation c: Moselfahrt, [77]) and a self-constructed dictation task consisting of 253 German words. Dyslexia was defined as a deficit in spelling ability as assessed with the R-T (at least 1.5 s.d. below the error rate expected from individual IQ) and a minimum of 60 misspellings in the self-constructed dictation task. Furthermore, a self-constructed computerized reading test ("Readspeed") indicated that dyslexic participants had a slower reading speed (silent reading, normal readers: mean 463 ms per word (s.d. 29 ms), dyslexic readers: 918 ms (122 ms), T(22) = 3.78, p < .01; reading aloud, normal readers: 612 ms (58 ms), dyslexic readers: 988 ms (109 ms), T(22) = 3.12, p < .01). In this test, subjects have to read a short story word by word either aloud or silent, and the response time to each word is measured. Only participants with an IQ of at least 110 were included in the study. All participants were native speakers of German. Exclusion criteria included poor educational opportunities, neurological disease or seizures, a self-reported history of attention-deficit hyperactivity disorder (ADHD) and auditory or visual impairments that might have interfered with reading skill acquisition. Eleven dyslexic subjects were recruited for participation (mean age: 24.9 yrs, range 19–30, 1 left-handed according to self-report, 1 woman). The 11 control subjects had a mean age of 26.1 yrs. (range 19–33, 2 left-handed, 1 woman) and were students at Hannover Medical School. There were no reliable differences in age, sex or handedness between dyslexics and controls. Mean IQ differed slightly between the two samples (controls: 135, range: 118–150, dyslexics: 126, range: 111–136; T(20) = 2.598, p < .0172). On average, control subjects had completed 16.3 yrs. of education compared to of 16.5 yrs for the dyslexics (T(20) < 1). Controls and dyslexics differed with respect to their spelling ability (R-T: 7.3 vs. 27.4 errors, T(20) = 7.7, p < .0001; dictation test: 19.8 vs. 98 errors, T(20) = 11.86, p < .0001). All participants received monetary compensation and gave informed consent to their participation. The study was approved by the ethics committees of Hannover Medical School and of the University of Magdeburg.

### Stimuli and procedure

Subjects participated in three different tasks that were conducted on different days in random order. On each day, several other ERP-paradigms were administered in addition (for details, see [33,34,66,78]. Each task comprised the subsequent presentation of two words on a computer screen. Each trial started with a fixation cross appearing for 500 ms in the center of the screen. The first word was then displayed for 200 ms and replaced by the fixation cross. Six hundred ms after the offset of the first word the second word was shown for 200 ms. The inter-trial interval (time from offset of the second word of a trial to the onset of the first word of the following trial) was randomly varied between 3000 ms and 4500 ms. Word length ranged from 3 to 12 letters. All words were written in yellow capital letters (1.5 × 0.9 cm) on a dark blue background and were viewed from a constant viewing distance of 90 cm. Response-times were measured from target-onset to response execution.

For the rhyme-judgment task (RJT), 240 word pairs (German nouns) that constitute a rhyme (e.g. Haus – Maus [house – mouse]) and 240 non-rhyme word pairs (e.g. Bett – Kind [bed – child]) were presented. Subjects had to indicate with a button press whether the two words constituted a rhyme or not. For the semantic judgment task (SJT), 240 semantically related (e.g. Gabel – Messer [fork – knife]) and 240 semantically unrelated word pairs (e.g. Blatt – Stern; [leave – star]) were constructed. Subjects had to indicate whether the two words were semantically related or not. In the third task 240 word pairs consisting of a definite article and a noun agreeing with respect to gender (der Hut [the hat; masculine gender]) and 240 additional word pairs with incorrect gender matching (das Chemie [the chemistry; neutral gender, correct gender in German is feminine]) were used. Participants had to indicate whether the word pair was grammatically correct or not (thus GJT = grammatical judgment task). Different words were used for all three conditions. Word frequency for the critical second words of the pairs ranged from 1 to 6413 occurences per 1 million words (Celex database [79]) and did not differ for the three tasks (RJT: mean frequency 220 occurences per 1 million words, SJT: 296, GJT: 226; F(2,238) < 1). In all three tasks, the word-pairs were presented in random order. A response button was positioned beneath each thumb. In each of the tasks, for six dyslexic as well as for six normal readers, the right button was used to signal a "yes" response and the left button was assigned to the "no" response. For the remaining subjects the order was reversed.

Importantly, for all three tasks different lists were created such that a given word appeared in the matching and non-matching conditions with equal probability. For example, in the semantic judgment task, in one scenario/HAUS/(house) served as the prime for an unrelated target like/AUTO/(car), in the second scenario it served as a prime for a related target/GEBÄUDE/(building), in the third it served as the target to an unrelated prime, and in the fourth scenario it served as the target to a related prime. Similar lists were constructed for the syntactic and rhyme judgment tasks. The choice of the actual scenario presented was counterbalanced across subjects.

### ERP-recording and data analysis

Subjects were seated in a comfortable chair placed in a sound attenuated and electrically shielded room and were instructed to relax. The EEG was recorded with tin electrodes mounted in an elastic cap (Electro Cap International, Eaton, OH) from 29 sites placed according to the International 10/20 system [80] referenced to an electrode located on the right mastoid. Horizontal eye movements were monitored with a bipolar montage using electrodes located on the outer ocular canthi of the left and right eye. Vertical eye movements were detected through electrodes located below and above the right eye. All channels were amplified using a 10 second time constant and processed with a bandpass filter between 0.01 and 100 Hz (half amplitude low and high frequency cut-offs), digitized at a rate of 250 Hz (AD resolution 12 Bit, 4 ms) and stored on a harddisk. Trials with eye-movement or blink artifacts were rejected using individualized amplitude criteria on the eye-channels by a computer routine. Briefly, a number of representative blinks of the particular subject were inspected and the rejection criteria were set such that blink artifacts were reliably rejected. In addition, routines for the detection of amplifier blocking were included.

ERPs were averaged separately for each subject, task, congruent, and incongruent target words for a time period of 1000 ms relative to a 100 ms prestimulus baseline. Only artifact-free trials with a correct response in the time-window 200 ms to 2500 ms after prime-onset were used for this procedure. Group averages were created by averaging the ERPs of all dyslexic and normal readers, respectively.

Difference potentials were computed for each task by subtracting point by point the potential evoked by congruent targets from that evoked by incongruent target words. These difference potentials reflect the neural correlates of semantic, phonological, and syntactic incongruency.

The data were evaluated using repeated measures analyses of variance (ANOVA) of the differences waves (incongruent – congruent condition). Factors used in the ANOVA were GROUP (dyslexic readers vs. normal readers; between subjects factor) as well as electrode factors as within subjects factors. Separate analyses were done for the midline (ML: Fz, Cz, Pz), parasagittal (PS: Fp1/2, F3/4, C3/4, P3/4, O1/2) and temporal (TE: F7/8, T3/4, T5/6) electrodes. In the ML analysis the electrodes yielded the three levels of an ANTERIORITY factor, while for the latter two sets electrodes were entered to yield an ANTERIORITY (PS: 5 levels, TE: 3 levels) and a LATERALITY (levels: left, right) factor. Arranging different analyses for the various electrode sets was preferred to including all sites into a single factor, since this option has less descriptive value regarding the topography of ERP-effects. Furthermore, to show the significance of the congruency effect proper, ANOVAs with the factors WORD TYPE (congruent, incongruent) and the electrode factors were computed separately for each task and for dyslexic as well as normal readers.

To assess topographical differences in the obtained ERP-effects between the RJT, SJT, GJT, and dyslexic and normal readers, we first performed the standardization of the data proposed by [81]. This is necessary because of non-linearity of signal conduction in the brain tissue and in the skull, ANOVA-models may confuse differences in the amplitude of an EEG-signal (due to differences in source strength) with genuine topographic differences. These standardized data were submitted to various ANOVAs. In all ANOVAs, the correction for non-spericity with the Huyhn-Feldt-epsilon coefficient was performed whenever applicable. Reported p-values are corrected.

N400 onset latencies and the onset latency of the anterior negativity in the GJT were computed by conducting one-sample t-tests against 0 for the difference waves for each group and each task. These were computed in the time-range 100 to 700 ms after stimulus presentation. Moving time-windows of 40 ms length were employed (i.e. the first time-window ranged from 100 to 140 ms, the second from 108 to 148 ms and so on). The middle point of the first time-window with a significant difference from 0 was taken as the onset latency, but only when at least three consecutive time-windows yielded a significant result. Furthermore, N400 onset latencies were computed and statistically evaluated with the jackknife-method as described by [82]. Here, a criterion of 20% of the maximum amplitude was used for determination of the onset and the differences waves were filtered (low-pass 8 Hz) prior to conducting the jackknife procedure. As these analyses yielded a virtually identical pattern to the approach described above, they will not be reported in detail in the results section.

## Abbreviations

ERP: event-related potential

RJT: rhyme judgment task

SJT: semantic judgment task

GJT: gender judgment task

RT: Reaction time

ANOVA: analysis of variance

WAIS-R: Wechsler Adult Intelligence Scale – Revised

HAWIE-R: Hamburg-Wechsler Intelligenztest für Erwachsene

R-T: Rechtschreibtest

## Authors' contributions

JR, SJ and TFM designed the study. PB acquired the data. JR analyzed the data. JR and TFM drafted the manuscript.
